# Reflections of Swedish Fathers in Late Adulthood on Their Past and Present Parental Role in Relation to the Mother

**DOI:** 10.1111/sjop.70079

**Published:** 2026-02-02

**Authors:** Maria Wängqvist, Nathalie Korhonen

**Affiliations:** ^1^ Department of Psychology University of Gothenburg Göteborg Sweden

**Keywords:** fatherhood, gender roles, late adulthood, lifespan development, parental roles

## Abstract

With a contextual and developmental perspective, this study aimed to examine Swedish late‐adult fathers' reflections on their past and present parental role in relation to the mother and to see how these reflections incorporate changes in gender and parenthood during recent decades in Sweden. Twenty Swedish fathers of adult children, aged 61–77 years, participated in an interview concerning their parental identity. Answers concerning their reflections on their parental role over time and in relation to mothers were analyzed using thematic analysis. We formulated themes concerning their (1) self‐positioning as a father in relation to the mother and gendered norms; (2) the fathers' wishes to have done things differently while expressing that they had no regrets over the choices they had made as a parenting couple; (3) alleviating regret by relating to history‐graded changes and societal and relational contexts; and (4) an experience of “growing together” as parenting partners, interpreted as the co‐construction of a joint parental identity, expressed either as a conflict‐free conversation climate around parenting being interpreted as agreement or as a joint understanding emerging through discussions about parenting issues. Analyses deepened the understanding of the traditional allocation of parental roles and how fathers, in light of their lifelong parenthood and current retrospective perspective, may wish they had done things differently, while simultaneously saying they had no regrets as their joint choices had made sense at the time and seeing the fact that the “kids are alright” as proof of their successful parenthood.

## Summary

- Fathers self‐positioned in relation to mothers and gendered norms, and integrated changing norms into their narratives of themselves as parents over time.
- Fathers' understanding of themselves as parents developed both through positioning themselves in contrast to mothers and through co‐constructing a parental identity within the parent dyad.
- Expressing a wish to have done things differently but having no regrets was interpreted through a life‐review perspective, making sense of earlier choices by situating them in context and considering that the children were doing well.

## 1 Introduction

In Sweden, as in many high‐income European societies, parenthood often involves two parents sharing responsibility and typically involves a lifelong commitment to the parental role and to the parent–child relationship. The way parents share responsibility, agree, and collaborate on parenting issues, and the extent to which they trust and support each other is referred to as co‐parenting (Feinberg 2003). In this study, we focus on how fathers make sense of their own parental role in relation to the mothers from a retrospective perspective of changing norms surrounding parents. In previous decades (1970–2010), societal changes have contributed to a more nuanced image of fathers' parenting, and much research has contributed to the science of parenting and gender roles (Lamb 2010). That the quality of fathers' and mothers' relationship affects fathers' engagement in parenting and father–child relationships has been a consistent finding (Kotila and Schoppe‐Sullivan 2015). Since the 1970s, governments of most developed economies have implemented policies to encourage working fathers to become more involved parents (OECD 2011). Sweden has been in the forefront of this development, making it a particularly salient context for the study of how these societal changes are incorporated in individuals' lived experiences and views of themselves as parents. Research has shown that children achieve better both socially and educationally when their fathers are more involved in their parental role and that more active and involved fathers express greater satisfaction with their lives and tend to have more stable relationships (Huerta et al. 2013). However, we still know more about mothers' parenthood than fathers'. Few studies have examined fathers' own experiences of parenthood (Olmstead et al. 2009), and even fewer from the lifespan perspective of fathers in late adulthood (Bates and Taylor 2012). Therefore, the focus of this study is on late‐adult fathers' reflections on their parental role in relation to the mother in the light of societal changes regarding gender and parental roles.

By asking fathers to look back on their parenthood from the perspective of late adulthood and as fathers of adult children, this study sets out to capture what aspects stand out as meaningful when viewed through a lifespan lens. The period of late adulthood is a significant aspect of this study as it adds a lifespan and developmental perspective to the research on fathers' parenthood. This period of life often entails a need to reflect on the time that has passed (Westerhof and Bohlmeijer 2014), which makes it relevant for the study of how fathers in late adulthood reflect on their parental role from this retrospective perspective and in relation to their current role as parents of adult children. Building on the unique perspective of fathers in late adulthood that have raised their children during times of changing norms surrounding fathers', this study aims to gain a deeper understanding of how they reflect on their past and present parental role in relation to the mother, and by doing so contribute to the knowledge regarding fathers' experiences. Even more so, these fathers' retrospective reflections are relevant from a historical and contextual perspective (see, e.g., Shwalb et al. 2013). The fathers in this study raised their children during decades of major societal transformations in how parents share responsibilities and in the Swedish context where these changes have been particularly salient. The study has an identity perspective, where identity is seen as the intersection between society and individuals' lived experiences (see, e.g., McLean and Syed 2015). The fathers' reflections on their parental role in relation to the mother have the potential to offer a window into how broader cultural shifts regarding gender and parenting practices have been incorporated into personal narratives, their parental identity.

### 1.1 Parental Roles and Gender

To understand how fathers in late adulthood reflect on their parental role in relation to the mother, we first address the influence of gender on parental roles and parenting practices in heterosexual parenting couples and how this has developed over time. A marker in the timeline for the development toward a more gender‐equal parenting occurred during the 1970s, when the idea took form that fathers should be more involved in their children's everyday life and care (Schoppe‐Sullivan and Fagan 2020; Suwada 2015). The 1970s saw the development of an attitude whereby closeness between children and their fathers was encouraged, thus reducing the focus on the man as the provider parent (Suwada 2015). In Sweden, there have been parallel developments in perceptions of gender‐stereotyped parental roles, with young adults (Trifan et al. 2014) reporting the change from fathers being decision‐makers and mothers being caregivers to both parents sharing decisions and garnering respect from their children. Thus, recent decades have witnessed a change from traditional and gendered views on parental roles to a more nuanced, less distinct father role compared with the mother role. These changes are evident both at a societal level in terms of policies and legislation and at the individual level as illustrated by Trifan et al. when describing shifts in perceptions and attitudes in relation to parental roles and gender.

The present study's participants (drawn from a longitudinal study; see, e.g., Hwang and Lamb 1997; Chuang et al. 2004; Wängqvist et al. 2022) became parents for the first time in the 1980s and spent their first years as parents during this decade. In Sweden, parental leave has been available since the 1970s, and from the start mothers and fathers had the right to the same number of days of parental allowance as well as the possibility to abstain from days, which would transfer to the other parent (Försäkringskassan 2021). Sweden's parental leave legislation is one of the most equal in the world, with parenting couples, since 2002, able to take 480 days off from work and receive 80% of their salary for the first 15 months of their child's life (Wells and Sarkadi 2012). According to Försäkringskassan (2021), among heterosexual parenting couples in Sweden in 1974, the mother took out 99.5% of all parental allowance days. Over the decades during which these participants have been parents, the development toward equal sharing of parental leave has been slowly progressing: In 2020, mothers used about 70% of the parental allowance days (Försäkringskassan 2021). Several laws have been introduced as an attempt to encourage parenting couples to share parental leave more evenly. Thus, Swedish policy and legislation strive to support gender‐equal parenting, and parenting practices have followed this direction, albeit at a slower pace and with more variability.

Eriksson and Brandén (2021) discussed why the development toward equality is slow: One argument concerned the workplace's role in maintaining a discourse on parental leave whereby maternal leave is expected and is seen as proof of a woman's parental involvement, while paternal leave is regarded as a choice and considered a privilege and an adaptable parenting level. Indeed, fathers' choices are highly affected by expectations and norms regarding men in the workplace (Eriksson and Brandén 2021; Haas and Hwang 2019) as well as the parent couple's financial situation (Eriksson and Brandén 2021). A continued effort toward equality in parenting has also been found in some studies from the US, where women and men who do not follow traditional gender roles can experience harassment in the workplace (Berdahl and Moon 2013). These studies illustrate the reciprocal interaction between individual parenting practices and norms in the workplace. Societal norms and views on parental roles are significant in parental couples' parenting practices and the interaction with these norms in parents' self‐positionings and reflections on their parental role are salient aspects of their parental identity. These findings that illustrate the changes in norms surrounding parenting and gender in Sweden during recent decades highlight the complexity of this development as it has not been entirely linear when it comes to parental roles and practices developing toward more gender equality. These historical and contextual aspects set the stage for the present study of fathers' reflections on their parental role in relation to the mother during these decades.

### 1.2 A Lifespan Perspective on Late‐Adult Fathers' Parenthood

This study takes a lifespan perspective, asking late‐adult fathers to reflect on their parental role from the time their first child was born until the present, their children now adults. In this study, the lifespan perspective is not only a theoretical framework but inherent in the retrospective design itself. In identity interviews we asked the fathers to revisit their parental role across time and to be open to reinterpreting it as they viewed it in retrospect. The lifespan perspective is relevant for the study in several ways. In its broadest meaning, a lifespan perspective involves the view of human development as a lifelong process, with researchers interested in stability and change in multiple domains (Baltes 1987). This perspective is also associated with a meta‐theory in which development is seen as multidirectional, plastic, embedded in culture and history, contextualized, and involving both gain and loss. Lifespan perspectives have highlighted how development is related to both normative age‐ and history‐graded influences as well as non‐normative ones (Baltes 1987; Baltes and Smith 2004). In relation to parental roles, these influences are relevant as parenthood is related to the historical and cultural context and the individual's development in terms of both normative age‐graded and non‐normative influences. From this perspective, parental roles may be viewed as developing in relation to the age of both parent and child; their historical and cultural context, for example the changing norms concerning the father role in recent decades; and non‐normative idiosyncratic influences, such as the quality of the parents' relationship, divorce, or illness. Building on this perspective, here we will describe salient developmental aspects of late adulthood, and the role of relationships in this time of life.

In developmental psychology, ages over 65 are often referred to as late adulthood (Whitbourne and Whitbourne 2019). Until middle age individuals tend to focus on the future and what is to come, while during late adulthood their focus tends to shift toward summarizing and putting their life into context (Westerhof and Bohlmeijer 2014). Individuals in late adulthood tend to have a more positive attitude toward life and a more positive autobiographical reminiscence than younger adults do (Agostino and Sheldon 2021), and are generally more attentive to emotional factors than younger adults are (Hess 2005). In late adulthood there is a tendency to remember more positive memories than negative ones (Westerhof and Bohlmeijer 2014; Kitayama et al. 2020). Studies have shown that these positive memories are linked to the years between 10 and 30 years old, as these years are central to one's identity development (Whitbourne and Whitbourne 2019). With regard to this study's focus on late‐adult fathers' reflections on their parental role across life, fathers may be focused on positive memories and silver linings, and the period of transitioning into parenthood (i.e., typically taking place around age 30) may be particularly salient in their retrospective reflections.

Another important aspect of late adulthood is how relationships change, especially for parents. As the parental role is often a salient aspect of an individual's identity, parents may see their children's development as a reflection of their own competence (Whitbourne and Whitbourne 2019). All types of relationships are important and essential to one's well‐being throughout life (Whitbourne and Whitbourne 2019). For older adults, the relationship between parent and adult child can play a vital role in the older adult's well‐being. It has been found that aging parents with good relationships are less likely to feel lonely or depressed (Whitbourne and Whitbourne 2019), and studies have shown that having satisfying relationships during later adulthood can decrease the risk of suffering from dementia as well as benefit one's cognitive functions (Charles and Carstensen 2008). Additionally, studies have examined the connection between men in late adulthood and close family relationships, with results indicating that men's well‐being is improved by having positive relationships with their family (Bates and Taylor 2012). The researchers found that involved grandfathers had fewer depressive symptoms and significantly higher scores on positive affect than disengaged grandfathers did. Another study showed that the time a grandparent spent babysitting their grandchildren was positively associated with their mental health (Condon et al. 2018). Thus, the parental role in late adulthood involves and is affected by both the parent–child relationship and the relationship with one's grandchildren.

A central part of aging is the developmental task of evaluating one's life (Westerhof and Bohlmeijer 2014). Engaging in such an evaluation of how life has been and the choices one is satisfied with as well as one's regrets is an important part of aging, as older adults often need to understand and evaluate the balance between their life's gains and losses (Filip et al. 2020). Individuals in late adulthood who have difficulty accepting certain losses and failures in their lives can get stuck ruminating and develop poor well‐being (Filip et al. 2020). These aspects, as well, were important to consider as we investigated fathers' reflections on their parental role over time and in relation to the mother. By highlighting how fathers can revisit earlier choices and identifications with understanding or regret, the study contributes to psychological perspectives on meaning making and identity integration in late adulthood.

### 1.3 The Present Study

The purpose of this study was to explore how Swedish fathers in late adulthood reflect back on their parental role in relation to the mother and how these reflections reveal the ways societal changes in gender and parental roles have been incorporated into their own parental narratives. At the time of data collection, the fathers in this study (who had previously taken part in a longitudinal study, starting in the 1980s when their first‐born child was 1–2 years old and ending their participation when the child was around 15) were in late adulthood and had adult children. These fathers, who have been parents during the recent decades of changing parental roles, particularly for men, were asked about their parenthood in retrospect, adding a developmental psychological perspective to the study. Thus, investigating these aspects from the position of late‐adult fathers of adult children integrated developmental and contextual (socio‐cultural as well as historical) perspectives that may contribute to a broader understanding of fathers' parenthood in relation to the mother.

## 2 Method

### 2.1 Participants and Recruitment

This study was conducted within the project Father Involvement from Early Childhood to Adulthood (FIECA). Data was collected during 2019/2020 and a total of 98 fathers participated in the project, having been recruited from the research project Gothenburg Longitudinal Study of Development (GoLD). The longitudinal project, initiated in 1981/1982 at the Department of Psychology at the University of Gothenburg, initially included 144 children aged 1–2 years and their parents (Lamb et al. 1988). The families, recruited from waiting lists for childcare in the Gothenburg area, came from different backgrounds and were representative of families in Gothenburg at the time (Broberg 1989). The children included in the project were the first‐born in the family, lived with both parents, and had not yet started preschool or family daycare when the study began. Data has been continuously collected for over 30 years through interviews, observations, and questionnaires. The fathers recruited for FIECA participated in the first six waves of the longitudinal project until their children, now in their early 40s, were 15.

Among the 144 fathers who took part in the first wave of GoLD, in 1981/1982, eight had chosen not to continue participating in the longitudinal study. Sixteen individuals who had previously participated chose not to participate in the data collection for FIECA, and seven were not reachable during the data collection. Additionally, 13 fathers who had previously participated in the longitudinal project had passed away, and two had serious health conditions or gave other reasons that made them unable to participate in the study. In total, 98 fathers participated in a background interview and survey within FIECA, and 93 of these participated in the interview used in this study. For this in‐depth qualitative investigation, a subsample of 20 interviews was used.

For this study, a sample of 20 fathers who had participated in an interview about their parental identity was selected for the thematic analysis of how fathers in late adulthood reflected on their past and present parental role in relation to the mother. The information in the interviews is extensive and dense, and it was deemed that 20 interviews was a suitable number for thematic analysis (Malterud et al. 2016), as the purpose of the method is to create a rich, detailed, and complex picture of the data whereby patterns and themes can be formed (Braun and Clarke 2006, with revisions Braun and Clarke 2019, 2021). These 20 interviews were selected from the larger sample following a randomized procedure where the only criterion was to make sure that they were not all from only one of the interviewers.

The 20 fathers selected for these analyses were aged 61 to 77 years (*M* = 68.3, SD = 4.4) at the time the interviews were conducted. The majority (*n* = 18) were in a relationship and living with their partner, 16 of them living with their (GoLD project) child's other parent (mother). Two fathers lived alone. The participants had two to four children (*M* = 2.5, SD = 0.8), and 19 of the 20 participants had grandchildren; those who had grandchildren had one to eight grandchildren (*M* = 3.8, SD = 2.0). Six participants were working, and 14 were retired, one of whom still worked to some extent. In Table 1 more detailed information from each participant is presented.

**TABLE 1:** Participants' answers to questions concerning their age, relationships, children and grandchildren, contact with oldest child, parental leave, and occupation in a structured background interview.

Participant	Age in years	Partner relationship (cohabiting or married)	Number of children	Number of grandchildren	How often do you have contact with your oldest child?	Do you see your child as often as you would wish?	Did you take parental leave?	Occupational status	Main occupation in life
Allan	61	Yes	2	4	Every day	Yes	Yes	—	Teacher
Bernt	71	Yes^a^	2	3	Once a week	Yes	No	Retired	Bank director
Bertil	73	Yes^a^	3	3	Once a week	No	Yes	Retired	Sales and personnel manager
Birger	66	Yes^a^	2	4	Every day	No	No	Retired	Florist
Björn	68	Yes^a^	2	4	A few times a month	Yes	Yes	Retired	Varied career
Börje	67	Yes^a^	2	3	Several times a week	Yes	Yes	Retired	Consultant (own company)
Christer	73	Yes^a^	2	3	Several times a week	Yes	Yes	Self‐employed	Construction manager
Christian	71	Yes^a^	4	6	Several times a week	Yes	Yes	Retired	Bank advisor
Dan	67	Yes^a^	2	3	Once a week	Yes	No	Retired	Group Manager
Edvin	63	Yes	3	6	Once a month	Yes	Yes	Self‐employed	Doctor
Einar	65	No	3	1	Once a week	No	Yes	Retired	Care coordinator
Erling	74	Yes^a^	4	8	Several times a week	Yes	Yes	Retired	Programmer/Systems Engineer/IT Development
Filip	63	Yes^a^	2	4	Several times a week	Yes	Yes	Working (employed)	Head of education
Folke	73	Yes^a^	2	6	Once a month	Yes	Yes	Retired	Music teacher
Gunnar	77	Yes^a^	4	6	Several times a week	No	No	Retired	Surgeon
Gustav	62	No	3	—	Once a week	No	Yes	Working (employed)	Carpenter
Göte	71	Yes^a^	2	4	Several times a week	No	Yes	Retired	Research and development
Ingvar	65	Yes^a^	2	2	Once a week	Yes	Yes	Working (employed)	Mechanical engineer
Kaj	66	Yes^a^	2	4	Several times a week	No	Yes	Retired	Senior physician and head of pharmaceutical company
Ulf	70	Yes^a^	2	3	A few times a month	Yes	Yes	Retired	Telecom administrator

^a^ In a relationship with first‐born child's mother.

### 2.2 Procedure

Before data collection, participants were informed about the study through a letter. The letter contained information about the project and conditions for participating and informed them that the research team would contact them about a week after the letter had been sent. The fathers were contacted by phone, and if they chose to participate, an interview was scheduled. The majority (*n* = 18) of the 20 interviews selected for this study were conducted at the Department of Psychology at the University of Gothenburg; one participant was interviewed at home, and one by phone. All interviews were conducted by either a male doctoral student or a female research assistant. Both interviewers were in their late twenties/early thirties; neither were parents at the time of the interview, but one of them was expecting. Both interviewers had Swedish as their native tongue. The interviewers were both employed in the project and were responsible for data collection and data management, including transcribing and organizing the data. After the data collection, the interviewers were involved in analyzing the material (for other studies than the present). The interviews were conducted in Swedish, and quotes from participants have been assigned pseudonyms and have to some extent been adapted for easier reading. The FIECA study has been approved by the Regional Ethics Review Board in Gothenburg (reference number 1014–18). The study was not preregistered, and data and study materials for the study are not publicly available, due to privacy and ethical restrictions.

### 2.3 Measures

#### 2.3.1 Background Interview

Through a structured background interview, all participants were asked questions about demographic, psychosocial, and relational topics. The questions from this interview that were relevant for describing the participants in this study were their occupation, relational status, living situation, and family.

#### 2.3.2 Parent Identity Interview

Marcia's identity status interview (Marcia et al. 1993) is a semi‐structured interview that was translated into Swedish and adapted to Swedish conditions within the longitudinal project (Frisén and Wängqvist 2011). Its section on parenthood was the basis for the qualitative analyses in this study. All interviews were based on a semi‐structured interview guide, with follow‐up questions varying depending on the participant's answers. The interview guide had specific questions asking about the mother, such as “In what ways is your role as a parent similar to or different from your child's other parent's role?”, “What do you think your child's other parent feels about your thoughts on parenting” and “In what ways has that affected you?” The fathers also talked about the mother in other parts of the interviews (i.e., where they were not directly asked about them), and these sections were also included in the analysis. Sections where these reflections were often shared were, for example, those asking about the father's thoughts about what had influenced their views on parenting and their parental role, who they talked to about these issues, and how they viewed their own parental role in the light of their children's current parental role (if their child had children on their own). Other sections that often involved answers of relevance to the study concerned those where the fathers were asked what advice they would offer themselves if they could go back, and if they had any regrets. Due to the identity status interview's developmental focus (Kroger et al. 2011), it was deemed appropriate for investigating how fathers talked about their parental role in relation to the mother from a developmental perspective. The length of the parenthood part of the interview varied from about 20 min to an hour, and the transcripts were 7–13 pages long. The fathers varied in how talkative they were in the interviews. Importantly, the length of the interviews was not the key factor for their density and how much they contributed to the analysis.

### 2.4 Data Analysis

Thematic analysis was judged to be appropriate for the study's purpose of investigating late‐adult fathers' reflections on their parental role in relation to the mother from a lifespan perspective and in the light of societal changes with regard to gender and parental roles. A thematic analysis enabled us to formulate and analyze patterns and themes within the material (Braun and Clarke 2006).

An inductive method, meaning that the analysis was based on data rather than theory (Braun and Clarke 2006), was used to get an in‐depth understanding of the fathers' answers in the interviews. The study took a critical realist position (Braun and Clarke 2021) from which the fathers' answers were viewed as representing their actual experiences, while also acknowledging that these accounts are inherently influenced by their subjectivity and context. The analysis was conducted on both a semantic and a latent level as the beginning of the coding process was more descriptive, with a transition through the coding process to interpretations beyond what was said. This approach meant that in our analyses we viewed the participants' descriptions as representations of their actual experiences, but we were attentive to the fact that these are always mediated by language, cultural positions, and contexts, including the interview setting and the longitudinal study having had a focus on fathers' relative parental involvement. The coding started on a semantic level for the analysis to represent the participants' own descriptions, but was developed toward a latent level with the purpose of identifying patterns that might have shaped the participants' descriptions. This dual approach means that we used different levels of depth in the analyses to elucidate both what was said and why this may be. We strived for a nuanced analysis that accounted for the participants' own stories and experiences while also contextualizing and interpreting the broader meaning that may have shaped these.

The phases of the thematic analysis were based on Braun and Clarke's (2006), with revisions (Braun and Clarke 2019, 2021) approach to thematic analysis: As a first step, NK read through the interviews about parenting in their entirety to find the parts that were relevant to the study's purpose. The fathers' answers varied in length: Some answered with a few sentences about a topic, while others gave more extensive answers. In the second step (2), the 20 interviews were coded inductively and closely to get an understanding of the fathers' subjective experiences of their parental role in relation to the mother. This was done by NK, who discussed extracts and codes with MW throughout the process. After the initial coding, the first draft of themes was developed by NK and discussed with MW. Third, more delimited themes were formulated, in which it was also specified what was not included in the themes based on the patterns identified. In this step we then moved beyond the semantic and descriptive level in the fathers' talk about their parental role in relation to the mother to formulate latent themes. This next step involved refining and reviewing the themes in relation to the coded extracts and ensuring that the latent meaning in the interviews was accurately captured. Finally, MW read through all interviews and coded them based on the defined theme structure to verify that it corresponded to the data. In this process, relationships between themes were formulated following the relational analysis add‐on technique for data integration (Robinson 2011), this contributed to the final definitions and names of the themes as they are described in the results.

## 3 Results

The thematic analysis was performed to get an in‐depth understanding of the participants' reflections on their parental role in relation to the mother from their retrospective position of late adult parents to adult children and in relation to societal changes across time. The themes and the relation between them are elaborated in the analysis below.

### 3.1 Self‐Positioning as a Father in Relation to the Mother and Gendered Norms

When fathers talked about their parental role in relation to the mother, they reflected on their own position relative to gendered parental roles and how they themselves had acted according to them. In this theme, the fathers described a traditional allocation of parental roles and identified themselves as fathers in position to the mother and the gendered norms surrounding men's and women's parenting. Those few participants who described another position than the traditional gendered parental role captured tension in relationships. These few fathers described ways of understanding their position in terms of personal characteristics and described ways to balance the tension in relation to the other parents' feelings.

Most fathers positioned themselves in line with a traditional allocation of parental roles in the parent dyad. This traditional allocation involved descriptions of mothers being more engaged, social, and communicative, and being the parent who had contact with the children and knew what was going on in their lives both now and in the past. Fathers whose answers were coded to this theme described their parental role as more practical, in the form of taking children places, taking part in activities, and focusing more on breadwinning and their work. The mother was described as the parent who was the most engaged and involved, whereas fathers described themselves as having more of a practical, supportive function. Filip spoke about their respective parental roles by saying “She's a pro, a pro at being a parent; so I get it, I'm probably the one who helps, assists… I'm more in the background, while she's the one who makes things happen.” Descriptions also involved the fathers' role in setting boundaries, which was contrasted with the mothers' “nice role” as described by the fathers themselves. For example, Christian talked about the parents' different attitudes toward worrying, saying “It's probably a bit of a mom syndrome, being careful. /…/ we men are probably a bit blunter.” This quote illustrates the fathers' gendered descriptions of mothers' roles as softer in terms of both boundary‐setting and worrying, and their positioning relating to a father role as stricter and more straightforward. This positioning was interpreted both as the role they had been ascribed and the role they had assumed within the parent dyad.

A couple of the fathers' descriptions did not include this traditionally gendered positioning of their parental role. Instead, they described how they had taken on a substantial part of parenting responsibilities, either continuously or increasingly over time, or how they had a closer and more emotional bond to their children. One participant (Allan) framed his descriptions of having to take on the overall responsibility for parenting due to the mothers' health and living situation as his having to be “not the mother but much more of a father.” He said “Yes, I had to do it all /…/ I got assigned everything as time went on, and the more I did the less did the other [the mother].” The other participant coded to this opposite position of the theme described himself as having a closer emotional bond and communication with his children and said this had been somewhat difficult to deal with in the parent dyad. He described his closer relationship to his children and how this affected the mother:

It's always been easier for me to keep in contact with them because I haven't put up a wall or created conflicts /…/ I don't think [partner's name] experiences the same contact. /…/ they call me more often, for example. It makes her sad sometimes, actually /…/ When [their daughter] calls I put it on the speaker, that works better. /…/ I know she feels a bit left out sometimes.

These participants were in the minority compared with the more common descriptions of a self‐positioning that aligned with traditional allocation of parental roles but nevertheless illustrate variability in fathers' parental identities.

### 3.2 A Wish to Have Done Things Differently but no Regrets

The second theme concerned the participants reviewing their parental role in retrospect in the light of contextual change with wishes to have done things differently but without expressing regret. The participants described that their choices made sense at the time even though they would have done things differently if they were parents today or if they could go back. This was interpreted as a way of expressing openness to different perspectives and changing norms and practices, while being compassionate and understanding toward themselves as parents when looking back through this new lens.

This theme was related to the theme concerning their self‐positioning as a father in relation to the mother and gendered norms, as it most often involved them reevaluating their allocation of parental roles in retrospect and in light of the mothers' current closer relationship with the children. Bernt describes his reasoning for not having regrets but still wishing to some degree he had done things differently: “To maybe be at home more; that is, not thinking so much about work, I could imagine, but it's not something that I regret exactly, because we were in agreement the whole time, my wife and I, that…it's okay that we divide it up.” Similar to Bernt, other fathers were (most often) clear about having made conscious choices that made sense at the time but reevaluated these choices from their current retrospective position. For example, Birger expressed that he and the mother had made conscious choices about him working more and her taking on more responsibility for their children. He said they had agreed at the time, but now, in retrospect, he wished he had been more present and worked less. Another aspect the fathers in retrospect expressed a wish to have done differently involved taking on the boundary‐setting parental role, but they also reasoned around the need for someone to take on this role and that it made sense for them to do it at the time. A third aspect concerned some fathers saying they wished they had taken parental leave and assumed a more active role in their children's everyday lives from early on. Dan talked about this, saying that he had been aware of the possibility but that it was not as common then as it is now. Thus, the fathers' retrospective reflections on what, from their current perspective, they wished they had done differently related to being present and less focused on work, being less strict and boundary‐setting, and being involved in their children's everyday lives from early on through parental leave.

Despite reasoning around these retrospective wishes to have done things differently, when specifically asked whether they regretted anything in their parenthood most of the fathers answered no. As a basis for their answer, they reasoned that their choices had made sense at the time, were understandable considering the context, and that the “kids were alright”. For example, Christian said “No, I probably feel like if I see how far they [the children] have come, how well they've done, then I don't feel like there's anything I want to change; they're both happy.” Furthermore, Erling explained why he had no regrets, saying “No such decisive things, no… no… I think we did a good job, you see.” Contrary, Ulf was the only father who expressed regret: “I should've been better; I feel now in retrospect like I didn't prioritize them [the children] as much as work. And I suffer a little from that now.” Thus, several fathers expressed that they wished they had done certain things differently, but to the specific interview question of whether they regretted anything in their parenthood, most answered no.

### 3.3 Alleviating Regret by Relating to History‐Graded Changes and Societal and Relational Contexts

The participants used different explanatory models to reason around their parental role and the decisions they had made as parents. These were often used to explain the gendered parental roles and concerned the cultural and relational context in terms of norms and expectations as well as the couple's own decision making. These explanatory models were seen as the fathers' way of making sense of their choices by situating them in time and context and interpreted as serving the purpose of alleviating regret in relation to their wish to have done things differently. Thus, this theme was related to the theme concerning a wish to have done differently as it may explain the lack of regret. Similar to the previous theme, it mainly concerned the fathers' self‐positioning as parents in alignment with traditional gender roles, and the fathers' reflections on the reasons why they had taken on the roles described in the theme concerning their self‐positioning. For example, Christer described being aware of their different roles but also said he, in retrospect, saw differences in how parents and society view the division of gender roles today, linked to societal norms:

No, I don't think we've had any major concerns about this [thoughts about parenthood], but what I've heard is just that I always went off to work and was gone, and she had to take care of sick children and stuff like that, but that… unfortunately, it generally burdens the female gender. We weren't quite as democratic; it's different today… What we did 30 years ago, it differs a lot, and legislation is different and everything, so… and then it was generally the woman who stayed home with sick children.

Like Christer's, all the descriptions coded to this theme involved a traditional allocation of parental roles, and one of the explanatory models that the fathers discussed related to their own upbringing having involved even more traditional parental roles. They described how their own parents had had gendered parental roles whereby the mother had not worked at all, and the father had been stricter than they themselves had been and had devoted much more time to work than they had. When the fathers described that they themselves had worked more than the mother had, they argued that an explanation for this was the influence from their own parents as role models. Another perspective that the fathers discussed as an explanatory model for their gendered parental roles was that they and the mother had mutually agreed on how to allocate parental responsibilities for financial and practical reasons, thus relating their decision to the relational context. These fathers reasoned about influences from macro‐contexts as well and mentioned societal norms and the support systems available throughout their parenthood. Several fathers whose responses were coded to this subtheme described their own children's division of parental responsibilities as an example of the societal changes that had occurred across these generations toward more equal parenting, and reflected on their own parental role in relation to their children's. Thus, when the fathers looked back on their own parental role compared with their own children as parents, they offered the explanatory model that societal norms surrounding parents had changed and situated these generational shifts within their own family. We interpret this as the fathers explaining their alignment with gendered parental roles by relating these to both macro‐contexts such as history‐graded changes and societal norms and micro‐contexts such as the parent dyad making joint decisions, and that they used these explanations as a way to alleviate regret and reinforce the compassionate understanding toward the choices they had made throughout their parenthood that was described in the previous theme.

### 3.4 Growing Together: Co‐Constructing a Joint Parental Identity

The third theme concerned the more micro‐level relational aspects of the parental identity and was formulated to capture an experience of *growing together* as parenting partners. Compared with the self‐positioned aspect of parental identity that we described in the first theme and that was created as opposite to the mother role, this process involved a shared parental identity and togetherness that had developed through implicit and explicit processes over time.

This growing together was interpreted from the fathers' use of *we* instead of *I* when asked about their parenthood. For example, when asked questions about parenthood in general that contained “you,” the fathers often answered “we” without much thought. We thus interpreted this “we” as an expression of the parental identity being shared and co‐constructed with the other parent. During the interviews, when asked “Was there something you wanted to convey to your children?”, some answered “We wanted to…” instead of “I wanted to…”. Dan, for example, answered the question regarding what values he wanted to convey to his children: “I think they've gotten what we wished for, the values, so to speak.” This excerpt highlights how fathers' experience of growing together as a parenting couple emerged in the interviews as a *we* instead of *I* parent. These types of answers gave the impression that the parents in the dyad influenced each other's ways of thinking about parental roles and parenting values more or less consciously.

The more explicit aspects of this theme consisted of two different positions through which a joint understanding was created. We viewed this as a dialectic between implicitly assuming an agreement by seeing conflict‐free communication as a sign of silent agreement, and openly discussing parenting issues within the parent dyad.

The first position involved statements by fathers relating to a silent agreement with the mother and involving them indirectly interpreting a conflict‐free communication climate around their parenting as agreement within the parenting couple. Filip, for example, answered the follow‐up question about whether the parenting couple had discussed values: “No, absolutely not; it was much more of a very natural process where things just happened. But somewhere there's a basic feeling of security, or a basic philosophy, both me and my wife, and we've lived by it.” These fathers talked more about the absence of conflicts around parenting issues than the presence of conversations. These descriptions indicated the belief that if there were no conflicts about parenting, this also meant that the parenting couple had the same values and agreed. An example of this was Bernt's description of the parenting couple having similar values, saying “Yes, I think so, yes, we don't argue about anything, about childrearing or anything; we agree on that, we're pretty much in agreement.” What Bernt described in the quote is an example of the statements that were coded as a type of silent form of communication within the parent couple whereby a lack of argument indicates agreement. Some fathers, like Filip, also described thoughts about how difficult it was to know who had come up with a certain thought or perspective related to parenting. Instead, it felt reasonable to assume that the other parent agreed and through the process they had come to be united in a shared parent identity.

In contrast to the silent communication being interpreted as agreement, there were fathers who said that their agreement with the mother had emerged from actively discussing issues and different opinions relating to parenting and parental roles. Björn described how talking about parenting issues with his partner had made him quite certain that they had similar views: “I do believe, we talk, we've talked together – she and I, we've probably talked a lot about it. And I think we have pretty similar views.” Thus, the descriptions coded to this part of the theme contrast with the descriptions involving silent communication. Instead of *growing together* through silently agreeing, these fathers talked about having discussed issues concerning their parenting and parental roles with the mother and how these discussions had helped them form agreements or discuss their differences and different opinions.

In contrast to this theme of growing together and the processes through which it could emerge, Allan described the experience of growing apart as parents and how conflicts and irreconcilable differences increasingly emerged and led to him and the mother leading separate lives both before and after their separation. Nonetheless, the overall theme concerned the co‐construction of a parental identity within the parent dyad, either through implicit assumptions or more explicit negotiations.

## 4 Discussion

This study of late‐adult fathers' reflections on their parental role in relation to the mother and in the light of changing norms surrounding fathers illustrates developmental processes involved in the reevaluation of a parental identity over time. The study contributes knowledge of how individuals in retrospect continue their development as parents by reevaluating the choices made from their current position and relationships and the ways in which they make sense of them. The study thus contributes knowledge on how parental identities evolve long after children are grown up, as well as the dynamics between individuals' lived experiences and socio‐cultural and relational influences across time.

The socio‐cultural changes that have occurred during the decades that these fathers have been parents were apparent in their reflections on their parental role. Traditionally, the father role has been that of breadwinner and provider (Lamb 2000), and these fathers aligned with this traditional allocation of parental roles. The analysis shows how these fathers positioned themselves in relation to gendered norms and incorporated the ways that they had shifted throughout their parenthood into their retrospective reflections of themselves as parents. These findings highlight the intersections between individuals' experiences and the socio‐cultural contexts in the fathers' views of themselves as parents, and as such it aligns with broader theories of identity development in relation to social contexts (i.e., theories of master narratives, McLean and Syed 2015). In addition to themes related to the broader socio‐cultural context, the analysis also showed how a shared parental identity could evolve over time within the relational context of the parent dyad. We interpret this as indicators of parental identity being constructed both through opposing self‐positions and through experiences of a sameness and togetherness within the parent dyad, thus illustrating a dialectic process and the ways in which this may be related to socio‐cultural changes and relational processes.

The study illustrates how the fathers' reflections on their parental roles are related to both normative and history‐graded influences, as these are described in theories of lifespan development (Baltes 1987; Baltes and Smith 2004). These influences were seen in the explanatory models the fathers used to make sense of the choices they had made as parents. The fathers related to normative influences in their explanatory models concerning societal norms and support systems. The history‐graded influences were apparent when they explained their choices in relation to their own parents as role models and compared them to their children as parents. The fathers relating to changes across generations and situating their own parental role within these could be seen as an indicator of these history‐graded influences being incorporated into their parental narratives. From the retrospective current position of the fathers, it was also clear how such influences may continuously change their meaning over time. That is, the fathers viewed their own parents in a different light now compared with when they first became parents, and from their current position contrasting their parental role to that of their children gave it a changed meaning. Non‐normative and more idiosyncratic influences were also described and were most apparent in the descriptions that were coded as comparatively different from the broader themes. Such influences could, for example, be related to fathers not describing a parental role that aligned with gendered norms and concerned aspects such as relational conflicts and divorce or parents' personalities. Most idiosyncratic influences that were described, such as explanatory models relating to the couple's financial situation as parents, for example, still aligned with the more normative influences of gendered parental roles.

Whereas the first and fourth themes concerned the development of the parental identity over time as seen in retrospect, the second and third themes seemed more clearly related to life‐review and the fathers revisiting previous choices relating to their parental role from their current position. Evaluating life and integrating one's life experiences is an important part of one's development in late adulthood (Westerhof and Bohlmeijer 2014), which may involve looking back at one's relationships in light of one's current position, as is illustrated in the study findings. In the descriptions coded to these themes, the fathers evaluated the roles they had assumed and made sense of them from their current perspective of fathers to adult children with a wish to have done things differently. They alleviated regret by situating their parenthood in relation to changing contexts across generations as well as to choices made for financial and practical reasons within the parent dyad. A particularly interesting finding was that fathers spontaneously brought up not taking parental leave as something they wished they had done differently. This finding is interesting as parental leave is one of the aspects that have changed during the past decades, but also because it is a comparatively short and early part of a child's and parent's life. That the fathers specifically described parental leave as something they would have done differently could indicate the salience of parental leave for the continued development of fathers' parental roles and relationships. Studies have shown that when parents share parental leave more equally between them, their child relates to the mother and father to the same extent (Almqvist and Duvander 2014). Broadly, the fathers' wishes to have done certain things differently could be related to them, from their current position, seeing the long‐term consequences and how this might have been related to their choices a long time ago. Contrary to the main pattern in the findings, one of the fathers in this study directly expressed regret at having worked too much, describing how he suffered from this in his later adulthood, wishing he had a closer relationship with his children. However, most of the fathers expressed little rumination or regret, even though they, in retrospect, were clear about their wish to have done things differently. We interpreted this as compassionate understanding and acceptance of their choices, which in turn could explain why they did not suffer from their wish to have done things differently. Part of the explanation for why they still wished to have done certain things differently may be related to their life stage, late adulthood, when people start evaluating what has been and integrating the advantages and disadvantages of their choices (Filip et al. 2020). Wishing one had done things differently may be a way of acknowledging that there are disadvantages to all choices, regardless of how much sense they made at the time. This process may be more of an intellectual or cognitive process and, as such, gentler than the more emotional process of regretting. These findings thus offer nuances to the understanding of life‐review that involve processes that help integration by making sense of both advantages and disadvantages of the choices one has made through life.

## 5 Limitations and Future Directions

In this study we used thematic analysis as it is suitable for investigating patterns in interview data. Twenty interviews can be considered a small sample to draw conclusions from; however, based on principles of information power (Malterud et al. 2016), the sample size was considered appropriate for the analysis as it allowed us to identify both semantic and latent patterns. Another aspect of the sample is that only fathers in Sweden participated, which means that the transferability to other countries may be low. Sweden is one of the world's most gender‐equal countries (Suwada 2015), which may have influenced how the fathers talked about parenting in relation to the mother. The participants are all in the same age range, from Sweden, and had their first child at approximately the same time. These aspects make the sample homogeneous. Even within such a sample there is variability, and questions and analysis were performed with an openness to various experiences of parenting and the parental role. Moreover, even though the fathers had various lifetime occupations, they all had established careers and most had a higher education. These types of careers might in turn be related to more sharing of parental responsibilities than what would be the case in a more diverse sample.

In a qualitative study one should always consider the impact of the researchers on the participants' interviews. This influence is both a strength and limitation. Anecdotally, we know that participants sometimes related to interviewers from their more experienced role as parents and talked in terms of offering advice. We do not have any systematic data about this, however, but believe that this senior position as someone “older and wiser” aligns well with the overall scope of the project. The age difference and the fact that the interviewers were not parents may, on the contrary, also have made some participants more hesitant to share their views on these issues. Moreover, their roles as participants in a longitudinal project in which their children were still currently participants might have impacted their answers. They might also have been aware of father involvement being a focus throughout the longitudinal project. The choice of semi‐structured interviews, in which the interviewer followed an interview guide and asked follow‐up questions, is also worth mentioning when discussing the study's limitations. While this procedure allowed the interviewer to follow up and adjust the interview to the participants' answers, not all questions were posed to everyone, which makes it difficult to interpret the findings in terms of how common the themes were (which, however, is not a purpose in thematic analysis). The interviews had a broader scope of investigating parental identity, of which the questions on parenthood in relation to the mother were only a part. Future studies could follow up the findings from this study with in‐depth interviews on specific aspects of the study findings, perhaps with fathers from different cultural contexts and generations.

## 6 Conclusions

This study examined reflections by fathers in late adulthood on their parental role in relation to the mother and in the light of changing norms surrounding fathers. Many of the findings can be understood in light of societal changes toward a more equal parenthood (in Sweden) during the decades when these fathers have been parents. The results illustrate how the fathers integrated such changes into their understanding of themselves as parents. As such, the study contributes knowledge on the intersections between self and socio‐cultural context in fathers' parental identities. The study is situated in a particularly salient cultural and historical context for investigating fathers' parental roles. From the unique experiences of these fathers who have been parents during times of changing parental norms, we learn how parents evolve their understanding of themselves over time and how this is done both through self‐positioning in opposition to mothers and co‐constructing a shared parental identity. Finally, the study illustrates how individual development in late adulthood involves family relationships being important for people's well‐being and that efforts to integrate and reevaluate past experiences are a salient developmental issue.

## Author Contributions

The second author conducted the initial coding and drafted the first version of the manuscript under the supervision of the first author. The first author reviewed all interviews, verified the thematic structure, contributed to refinements of the analysis, and revised the manuscript. Both authors contributed to the development of the final themes and approved the final version of the manuscript.

## Funding

This work was supported by Forskningsrådet för Hälsa, Arbetsliv och Välfärd, 2016‐00529. Grants from the Swedish Research Council for Health, Working Life and Welfare supported this research.

## Ethics Statement

This research was approved by the Regional Ethics Review Board in Gothenburg (reference number 1014–18). Participants filled out a written informed consent before data collection.

## Conflicts of Interest

The authors declare no conflicts of interest.

## Data Availability

The data are not publicly available due to privacy and ethical restrictions.
